# A pre-and-post study of an urban renewal program in a socially disadvantaged neighbourhood in Sydney, Australia

**DOI:** 10.1186/1471-2458-12-521

**Published:** 2012-07-12

**Authors:** Bin Jalaludin, Michelle Maxwell, Basema Saddik, Elizabeth Lobb, Roy Byun, Rodrigo Gutierrez, John Paszek

**Affiliations:** 1 Centre for Research, Evidence Management and Surveillance, South Western Sydney Local Health District, Sydney, Australia; 2 School of Public Health and Community Medicine, University of New South Wales, Sydney, Australia; 3 Population Health Directorate, South Western Sydney Local Health District, Sydney, Australia; 4 College of Public Health & Health Informatics, King Saud bin Abdulaziz University for Health Sciences, Riyadh, Saudi Arabia; 5 Department of Family & Community Services - Housing NSW, Sydney, Australia

**Keywords:** Urban renewal, Pre-and-post study design, Socio-economic disadvantage, Social housing, Evaluation

## Abstract

**Background:**

Urban renewal programs aim to target both the physical and social environments to improve the social capital, social connectedness, sense of community and economic conditions of residents of the neighbourhoods. We evaluated the impact of an urban renewal program on the health and well-being of residents of a socially disadvantaged community in south-western Sydney, Australia.

**Methods:**

Pre- and post-urban renewal program surveys were conducted with householders by trained interviewers. The urban renewal program was conducted over 16 months and consisted of internal upgrades (including internal painting; replacement of kitchens, bathrooms and carpets; general maintenance), external upgrades (including property painting; new fencing, carports, letterboxes, concrete driveways, drainage and landscaping), general external maintenance, and social interventions such as community engagement activities, employment initiatives, and building a community meeting place. The questionnaire asked about demographic characteristics, self-reported physical activity, psychological distress, self-rated health, and perceptions of aesthetics, safety and walkability in the neighbourhood. We used the paired chi-square test (McNemars test) to compare paired proportions. A Bonferroni corrected p-value of <0.0013 denoted statistical significance.

**Results:**

Following the urban renewal program we did not find statistically significant changes in perceptions of aesthetics, safety and walkability in the neighbourhood. However, post-urban renewal, more householders reported there were attractive buildings and homes in their neighbourhood (18% vs 64%), felt that they belonged to the neighbourhood (48% vs 70%), that their area had a reputation for being a safe place (8% vs 27%), that they felt safe walking down their street after dark (52% vs 85%), and that people who came to live in the neighbourhood would be more likely to stay rather than move elsewhere (13% vs 54%). Changes in psychological distress and self-rated health were not statistically significant.

**Conclusions:**

We found an increase, in the short-term, in the proportion of householders reporting improvements in some aspects of their immediate neighbourhood following the urban renewal program. It will be important to repeat the survey in the future to determine whether these positive changes are sustained.

## Background

Neighbourhood contextual factors can influence the physical, social and mental well-being of residents.[1-8] Improving and enhancing neighbourhoods may, for example, promote a healthier lifestyle that includes increased opportunities for physical activity[9-11] and better access to healthy foods.[12]

Similarly, environmental design and layout can influence social interactions and the level of social capital and cohesion in a community.[13-15] Neglected urban environments have been linked to social isolation and depression.[6,16] The provision of decent housing, safe playing areas, transport, green spaces and street lighting makes the community feel good about a place and leads to greater levels of interaction and community participation.[17]

Urban renewal or regeneration programs aim to target both the physical and social environments including improvements to housing, landscaping of the immediate neighbourhood, increasing safety and improving employment opportunities.[18] These measures are thought to improve the social capital, social connectedness, sense of community and economic conditions of residents of the neighbourhoods. There are only a few studies that have sought to evaluate health outcomes and general well-being of residents as a result of urban renewal[19-23] and there has been only one study from Australia.[18] A synthesis of evaluations of the United Kingdom national renewal program found little evidence of the impact of the national renewal program on socioeconomic or health outcomes.[24]

We aimed to evaluate the impact of an urban renewal program on the health and well-being of residents of a socially disadvantaged community in south-western Sydney, Australia. The objectives of the study were to assess the relationship between urban renewal and perceptions of the neighbourhood, to evaluate whether urban renewal influences social capital and to assess the relationship between urban renewal and perceived health status.

## Methods

### Study design

We used a ‘pre and post’ intervention study design to evaluate the impact of the urban renewal program. The pre-intervention survey was conducted over a four month period from December 2008 to April 2009. The urban renewal program, or intervention, was conducted between April 2009 and August 2010. The post-intervention survey was conducted between April 2011 and May 2011, about eight months following the completion of the urban renewal program. We obtained ethics approval for this study from the Sydney South West Area Health Service (Western Zone) Human Research Ethics Committee.

### Study site

The study site was two streets of established social housing in a fringe suburb about 45 kilometres to the south-west of the Sydney central business district. The style of housing was based on the Radburn design where townhouses were built around long cul-de-sacs, often centred on a park, with back fences facing the street. The area around the study site (population = 882) is severely socio-economically disadvantaged. From the 2006 Census, compared to the suburb (population = 9,695) and Sydney (population = 4.1 million), the area around the study site has a younger population (38%, suburb = 33%, Sydney = 24% less than 18 years of age), a higher unemployment rate (19%, suburb = 8%, Sydney = 5%), fewer with a tertiary qualification (25%, suburb = 31%, Sydney = 43%), a greater proportion who did not own a motor vehicle (27%, suburb = 8%, Sydney = 13%) and a higher proportion of social housing tenants (52%, suburb = 12%, Sydney = 5%) (http://www.censusdata.abs.gov.au/; accessed 2 May 2012).

A community safety audit conducted about six months prior to the pre-intervention survey of this area found high employment and low income levels, many single parent households, high levels of graffiti, vandalism, and crime, high levels of dissatisfied tenants, poor lighting and poor maintenance of public areas. The community audit was conducted over an afternoon and evening on one day by a team that included residents, and representatives from local government, the New South Wales Department of Housing and the local police.

### Recruitment of study subjects

All households in the two streets (n = 57 households) were invited to participate in the study. Each household was sent a letter by the New South Wales Department of Housing which explained the aims of the study and invited their participation. The letter also informed that the household would be contacted by a market research company to conduct the face-to-face survey. Only those householders who had completed the pre-intervention survey were invited to participate in the post-intervention survey.

### Questionnaire survey

The market research company conducted both the pre- and post-intervention surveys. Trained interviewers from the market research company doorknocked the households. At the first point of contact with an adult member of the household, the interviewer introduced the study and explained the aims of the study. If the householder was willing to participate, the interviewer then obtained informed consent for the study and advised the householder that institutional ethics approval had been obtained.

The survey took approximately 40 minutes to administer and measured sense of community, social capital, neighbourhood participation and self-reported indicators of physical and mental health. Specifically, the questionnaire asked about demographic characteristics, smoking and hazardous alcohol consumption, self-reported physical activity, psychological distress, self-rated health, and perceptions of safety, aesthetics and walkability in the immediate neighbourhood or local area. Questions around social connectedness, social capital, self-rated health, psychological distress and health risk factors were from the New South Wales Population Health Survey (http://www.health.nsw.gov.au/PublicHealth/surveys/questionnaire_adult.asp; last accessed 14 May 2012). Questions about perceptions of the neighbourhood safety, aesthetics and places for walking were from the validated Neighborhood Environment Walkability Scale (NEWS) (https://www.activelivingresearch.org/node/10649; last accessed 14 May 2012). Terms such as the ‘immediate neighbourhood’ or ‘local area’ refer to the immediate area around the two streets.

Only householders over 18 years of age who were able to answer the questionnaire in English were eligible for participation in the face-to-face survey. Up to five attempts were made to contact the household and household visits were made on different days (including weekends) and at different times of the day to ensure a high success rate in contacting the household. Once the household had been contacted and the householder was willing to participate in the study, the interview was conducted at that time or at another time convenient to the householder. Similar procedures for recruitment and data collection were followed for both the pre- and post-intervention surveys.

### The intervention

The urban renewal program - ‘the intervention’ - was conducted over a 16 month period and consisted of:

· Internal upgrades which included internal painting, replacement of kitchens, bathrooms and carpets where required, and general maintenance such as repairing water leakages, faulty windows and doors;

· External upgrades which included property painting, new front and back fencing, new carports, letterboxes, concrete driveways, drainage, landscaping, as well as general external maintenance such as repairs to roofs; and

· Social interventions such community engagement activities (for example, street picnics, family fun days, community newsletter), learning and employment initiatives (for example, conducting training courses and employment transition programs), and establishing a community meeting place to conduct community programs and activities.

### Data analysis

Body mass index (BMI) was calculated from self-reported weight and height and was categorised into underweight (BMI < 18.5), healthy weight (BMI 18.5-24.9), overweight (BMI 25–29.9) and obese (BMI 30+). In our study, adequate physical activity was defined as a total of 150 minutes per week. Total minutes were calculated by adding minutes in the last week spent walking (continuously for at least 10 minutes), minutes doing moderate physical activity, plus twice the minutes doing vigorous physical activity. Hazardous alcohol drinking was defined as consumption of more than two standard drinks on any one day [25]. Psychological distress, measured using the Kessler Psychological Distress Scale (K10), was categorised as follows: low to moderate < =21, high to very high = 22 + .

Data from the pre- and post-intervention questionnaires were analysed using SAS v9.2 (SAS Institute Inc., Cary, NC, USA). Only residents who had completed both the pre- and post-intervention surveys were included in the analysis. All findings from the survey are presented in the Results. We used the Fisher-Freeman-Halton exact test (StatsDirect v2.7.8, Chesire, UK) to compare independent proportions and the paired chi-square test (McNemars test) to compare paired proportions (SAS v9.2, SAS Institute Inc., Cary, NC, USA). A p-value of less than 0.0013, after applying the Bonferroni correction for multiple testing (n = 39 tests), denoted statistical significance. Uncorrected exact p-values are presented throughout the manuscript.

## Results

In the pre-intervention survey, 42 householders completed questionnaires out of the 57 eligible households (response rate 74%). In the post-intervention survey, 28 of 29 eligible householders completed the questionnaires (response rate 97%). Thirteen householders from the pre-intervention survey had left the study site and were therefore ineligible for the post-intervention survey and one eligible householder did not consent to participate.

There were no significant differences in any of the demographic characteristics between the group who participated in both the pre- and post-intervention surveys (n = 28) and the group who participated only in the pre-intervention survey (n = 14) (Table 1).

**Table 1:** **Characteristics of participants completing both pre- and post-intervention surveys and participants completing only pre-intervention survey**^**1**^

		**Completed pre- and post-intervention surveys**		**Completed pre- intervention survey only**		**p-value**
		**N = 28**		**N = 14**		
		**n**	**%**	**n**	**%**	
Age (years)	18-34	10	36	7	50	0.72
	35-54	14	50	5	36	(3df)
	55-74	3	11	2	14	
	75+	1	4	0	0	
Sex	Male	8	29	2	14	0.45
	Female	20	71	12	86	
Education level	Not completed year 12	21	75	8	57	0.31^2^
	Completed year 12	2	7	2	14	
	TAFE	1	4	4	29	
	University	3	11	0	0	
	Other	1	0	0	0	
Employment	Working	4	14	6	43	0.06
status	Not working	24	86	8	57	
Country of birth	Australia	22	79	12	86	0.70
	Other	6	21	2	14	
Language spoken	English	25	89	12	86	>0.99
at home	Non-English	3	11	1	7	
	Refused	0	0	1	7	
Aboriginality	Yes	6	21	3	21	>0.99
	No	22	79	11	79	
Length of time	<=5	13	46	10	71	0.40
lived in	6-10	6	21	1	7	(4df)
neighbourhood	11-15	3	11	0	0	
(years)	16-20	4	14	1	7	
	>20	2	7	2	14	
	Mean (SD^3^)	8.2		6.9		
		(6.85)		(8.09)		
Number of people	1-3	19	68	12	86	0.22
living in household	4-7	9	32	2	14	
Living with	Alone	5	18	2	14	>0.99
	Partner	1	4	0	0	(4df)
	Children	16	57	8	57	
	Partner and children	5	18	3	38	
	Other family	1	4	1	7	
Number of	None	9	32	5	36	0.36
children <16 years	1-3	12	43	8	57	(2df)
in household	4+	7	25	1	7	
Number of motor	0	13	46	5	36	0.51
cars in working	1+	15	54	9	64	
order in household						

^1^Includes participants who were ineligible because they had left the two streets (n = 13) or who refused to participate in the post-intervention survey (n = 1); ^2^Did not complete year 12 vs rest; ^3^SD = standard deviation.

Of the 28 householders who completed both pre and post-intervention surveys, 71% were women, 86% were aged 18–54 years, 89% spoke English at home, 82% had not completed year 12, 57% were the heads of single parent families, 86% were not working for income and 46% did not own a motor vehicle.

Perceptions of safety and aesthetics of the immediate neighbourhood are presented in Table 2. Following the urban renewal program, a greater percentage of householders reported that they felt safe walking from the bus or train at night and a smaller proportion reported that that the level of crime made it unsafe to walk in the immediate neighbourhood in the daytime. There were no significant statistical differences in any of the reported perceptions about neighbourhood safety. Many more of the householders responded positively to questions around the aesthetics of the immediate neighbourhood (Table 2). This was particularly for questions around pleasant natural features, attractive buildings and homes, and the immediate neighbourhood being free of graffiti, rubbish and litter. However, none of these were statistically significant

**Table 2:** Perceptions of neighbourhood safety, aesthetics and places for walking (N = 28)

	**Pre-intervention**		**Post-intervention**		**p-value**
		**n**	**%**	**n**	**%**	
*Neighbourhood safety (Somewhat/strongly agree)*					
Streets in my local area are well lit at night	16	57	16	57	1.00
There is a lot of petty crime in my local area (eg. vandalism, shoplifting)	27	96	25	89	0.63
There is a lot of major crime in my local area (eg. armed robberies, break-ins, attacks)	17	61	17	61	1.00
The level of crime in my local area makes it unsafe to go on walks during the day	5	18	2	7	0.45
The level of crime in my local area makes it unsafe to go on walks at night	19	68	21	75	0.75
I would feel safe walking home from a bus or train stop at night	15	54	22	79	0.093
*Neighbourhood aesthetics (Somewhat/strongly agree)*					
There is lots of greenery around my local area (trees, bushes, household gardens)	22	79	25	89	0.51
There is tree cover or canopy along the footpaths in my local area^1^	10	37	17	63	0.059
There are many interesting things to look at while walking in my local area	4	14	5	18	1.00
My local area is generally free from litter	4	14	9	32	0.23
My local area is generally free from rubbish (old furniture, broken glass, etc.)	6	21	11	39	0.27
My local area is generally free from graffiti	5	18	12	43	0.12
There are attractive buildings and homes in my local area	5	18	18	64	0.0072
There are pleasant natural features in my local area (eg. nature reserves, hills, lakes)	14	50	10	36	0.070
*Places for walking (Somewhat/strongly agree)*					
There are footpaths on most of the streets in my local area	17	61	14	50	0.63
The footpaths in my local area are well maintained^1^	13	48	15	56	0.77
There is a grass strip that separates the streets from the footpaths in my local area^1^	20	74	23	85	0.51
The footpaths in my local area are separated from the traffic by parked cars	15	54	14	50	1.00
There are bicycle/walking paths in or near my local area that are easily accessible^1^	15	54	16	59	1.00
There is a park or nature reserve in my local area that is easily accessible	14	50	16	57	0.77

^1^missing = 1

After the urban renewal program, more householders reported being satisfied with their neighbourhood as a place to live and felt that they belonged to the neighbourhood (Table 3). More householders also reported that their area had a reputation for being a safe place and they felt safe walking down their street after dark in the post-intervention survey.

**Table 3:** Perceptions of the neighbourhood connectedness and social capital (N = 28)

	**Pre-intervention**		**Post-intervention**		**p-value**
	**n**	**%**	**n**	**%**	
*Neighbourhood connectedness (Strongly agree/agree)*					
Most people can be trusted	16	57	13	46	0.58
I feel safe walking down my street after dark^1^	14	52	23	85	0.0039
My area has a reputation for being a safe place^2^	2	8	7	27	0.13
I feel as though I belong to this neighbourhood^1^	13	48	19	70	0.15
I am satisfied with my neighbourhood as a place to live^1^	17	63	20	74	0.55
*Social capital (Yes)*					
People who come to live in this neighbourhood stay for a number of years rather than tend to move on^3^	3	13	13	54	0.013
Sad if have to leave the neighbourhood^2^	13	50	17	65	0.42
Volunteered in the last 3 months	10	36	10	36	1.00
Attended a community event in the last 6 months	16	57	16	57	1.00
Active member of a local organisation	12	43	10	36	0.77
Visited someone in neighbourhood in the last week	21	75	21	75	1.00
Run into friends at the local shops	24	86	28	100	0.13

^1^missing = 1; ^2^missing = 2; ^3^missing = 4

Following the urban renewal program, a greater proportion of householders reported that people who come to live in the neighbourhood would be more likely to stay on for a number of years rather than move elsewhere (Table 3). More householders also reported that they would be sad to leave the neighbourhood.

There were no significant differences in the proportion of daily smoking, hazardous alcohol intake, adequate physical activity, and overweight/obesity before and after the urban renewal program (Table 4).

**Table 4:** Health behaviours, health status and health service utilisation (N = 28)

	**Pre-intervention**		**Post-intervention**		**p-value**
	**n**	**%**	**n**	**%**	
*Health behaviours*					
Daily smoker	17	61	15	54	0.79
Hazardous alcohol intake	13	46	14	50	1.00
Adequate physical activity	16	57	14	50	0.79
*Health status*					
Overweight/obesity	14	50	15	54	1.00
‘Excellent/very good/good’ self-rated health	18	64	14	50	0.34
‘High/very high’ psychological distress^1^	11	41	7	26	0.39
*Health service utilisation*					
Visited general practitioner at least once in the previous 4 weeks	17	61	14	50	0.55

^1^missing = 1

Although fewer householders reported ‘high/very high’ psychological distress following the urban renewal program (pre-intervention = 41%; post-intervention = 26%), this change was not statistically significant. There were also no statistically significant changes in the proportion of householders reporting ‘excellent, very good, good’ health, and in visits to a general practitioner in the previous four weeks (Table 4).

## Discussion

This study was undertaken to evaluate the effectiveness of an urban renewal program in a social housing neighbourhood in south-western Sydney, Australia. We did not find statistically significant changes in perceptions of aesthetics, safety and walkability in the neighbourhood or in health status following the urban renewal program. We did, however, observe statistically non-significant increases in the proportion of householders reporting that there were attractive buildings and homes in the immediate neighbourhood, that they felt safe walking down their street after dark and that people who come to live in the neighbourhood would be more likely stay on for a number of years rather than move elsewhere. We also found, although not statistically significant, an increase in the proportion of householders reporting increased connectedness to their neighbourhood and fewer householders reporting ‘high/very high’ psychological distress following the completion of the urban renewal program.

Results from the few published studies are mixed. In a pre-and-post study of 98 households in a deprived area with sub-standard housing in Newcastle, United Kingdom, where the neighbourhood intervention was similar to ours (environmental improvements, external fabric repairs, refurbishment, some demolition of empty dwellings, renovation grants for individual dwellings and security and road safety improvements),[23] a post-intervention survey showed significantly fewer mental health problems but also a significantly greater proportion reporting ‘not good’ health. There were also significantly larger proportions of participants who reported that their area was a very/quite nice place to live and very/quite safe.

It is suggested by Blackman et al.[23] that improvements in mental health in their study may be due to the perceptions of increased neighbourhood safety although the authors acknowledge that improvements in mental health could also influence perceptions of safety. The link between urban renewal, perceptions of neighbourhood safety and mental health is not clear. Petticrew et al.[22] investigated the health and well-being of people provided with new social housing in the United Kingdom and found there was no change in the mean mental health score despite improved perceptions of neighbourhood problems such as vandalism and graffiti, general appearance of the area and adequate street lighting. On the other hand, in a prospective controlled study of the health impacts of urban renewal, Thomson et al.[20] compared 50 households who moved to newly built housing in the same locality with 50 control households from a matched nearby locality and found no difference in the mean scores of both the mental health component of the SF-36 and perceptions of neighbourhood problems such as vandalism, litter and rubbish, crime, adequate street lighting.

The mixed results for mental health are especially of interest. Both people and places can impact on mental health. There are links between the social environment and mental health[26-28] and also between social capital and mental health.[29] Although, like us, Blackman et al.[23] showed improvement in mental health after the urban renewal program, others have not been able to demonstrate this.[20-22] The most likely explanation is that compositional factors, which also have important influences on health,[30] are not, or are inadequately, addressed by urban renewal programs. Residents of neighbourhoods targeted for urban renewal often suffer from multiple deprivations, including poor housing conditions, and any interventions to improve health and well-being should also target other social determinants of health, for example, education and unemployment.

We did not find any changes in health behaviours (daily smoking, hazardous alcohol consumption, adequate physical activity), health status (BMI, self-rated health) or use of health services (visits to a general practitioner) following the urban renewal program. This is not surprising as the urban renewal program was not specifically targeting health behaviours and health status and the follow-up period was very short (eight months). Only one other study has reported the effects of an urban renewal program on health behaviours and health service utilisation. In a much larger study than ours (n = 166 adults with paired data five years apart), Blackman et al.[23] reported mixed results post-housing renewal with some deterioration of general health status, increase in chronic respiratory symptoms, but improvements in mental health problems and no overall change in health service utilisation. However, they reported a marked decline in smoking rates (72% to 28%) and suggest that this could be partly attributable to improvements in mental health.

There has only been one published study from Australia on the impact of urban renewal. Kelaher et al.[18] evaluated a place-based whole of government intervention to narrow the gap between disadvantaged neighbourhoods and the rest of the state of Victoria, Australia. In this ‘pre-and-post’ study design, there was increased reporting of ‘good/very good/excellent’ self-rated health and satisfaction with life following the intervention. In contrast we found we found that fewer residents rated their self-rated health as ‘good/very good/excellent’ (decrease from 64% to 50%). Internationally, the effects of urban renewal on self-rated health have also been mixed. Petticrew et al.[22] reported significantly better self-rated health following an urban renewal program whereas Thomson et al.[20] reported no difference and Huxley et al.[21] reported a decrease in self-reported ‘satisfaction with health’. The reasons for such mixed results are likely due to the fact that the relationships between housing, deprivation and health are complex.

Our study has a number of limitations. The major limitation is our small sample size which limited our ability to detect small, yet meaningful, differences pre- and post-intervention. Despite this, our results were consistent with other published studies that have evaluated urban renewal or regeneration programs. The other major limitation was that we did not have a comparison group to take into account any changes that would have occurred due to factors other than the urban renewal program. Further, longer follow-up is required to determine whether changes are sustained over the longer term, and to be able to detect changes in health-related behaviours. We conducted a large number of statistical tests, and although we corrected for multiple testing, we must exercise caution when interpreting the results. Finally, our study was conducted in a social housing neighbourhood and therefore our results may not be generalisable to similar urban renewal interventions in non-social housing neighbourhoods.

The urban renewal intervention in this study consisted of a number of initiatives and included physical improvements to the homes (both internally and externally), improvements to the immediate physical neighbourhood environment (for example graffiti removal and landscaping) and social interventions such as community activities, learning and employment initiatives and establishing a community meeting place. All of these initiatives would have been likely to contribute to the householders’ perception of their immediate environment and their health status. It is not possible to ascribe changes in perception of the immediate neighbourhood to any one component of the urban renewal program.

The urban renewal intervention did not specifically target health risk factors such as smoking, hazardous alcohol consumption, physical activity and mental health. This may be one of the reasons we did not see corresponding changes in health risk factors over the study period. Also, changes to health risk factors are usually only observed over a longer time period than the current study allowed. We anticipate that the urban renewal program together with community development initiatives and specific interventions to address health risk factors will lead to long-term increases in social capital, health and well-being.

## Conclusions

We conducted an evaluation of an urban renewal program in a socially disadvantaged neighbourhood in south-western Sydney, Australia. The post-intervention survey was conducted about eight months after the completion of the urban renewal program. In the short-term there were many positive changes in perception of the immediate neighbourhood. However, as a synthesis of evaluations of the United Kingdom national renewal program found little evidence of the impact of the national renewal program on socioeconomic or health outcomes[24], it will be important to repeat the survey in the future to determine whether these positive changes are sustained.

## Competing interests

The authors declare that they have no competing interests.

## Authors’ contribution

BJ, MM, BS and EL conceived, designed and conducted the study and drafted the manuscript. RG and JP provided input into the questionnaire, study design and the draft manuscript. RB provided input into data interpretation and the draft manuscript. All authors read and approved the final version of the manuscript.

## Pre-publication history

The pre-publication history for this paper can be accessed here:

http://www.biomedcentral.com/1471-2458/12/521/prepub
